# Altered miRNA expression in the lesions of cutaneous leishmaniasis caused by *L. major* and *L. tropica* with insights into apoptosis regulation

**DOI:** 10.1038/s41598-025-03802-1

**Published:** 2025-07-01

**Authors:** Taha Masoudsinaki, Shima Hadifar, Hamzeh Sarvnaz, Mohammad Farahmand, Nasrin Masoudzadeh, Vahid Mashayekhi Goyonlo, Mohammadali Kerachian, Reza Erfanian Salim, Mourad Barhoumi, Seyed Latif Mousavi Gargari, Hossein Heydari, Sima Rafati

**Affiliations:** 1 Department of Immunotherapy and Leishmania Vaccine Research, Pasteur Institute of Iran, Tehran, Iran; 2 Department of Cell Biology, Faculty of Basic Sciences, Shahed University, Tehran, Iran; 3 Pediatric Infectious Disease Research Center, Tehran University of Medical Sciences, Tehran, Iran; 4 Cutaneous Leishmaniasis Research Center, Mashhad University of Medical Sciences, Mashhad, Iran; 5 Noor Eye Hospital, Tehran, Iran; 6 Molecular Epidemiology and Experimental Pathology (MEEP)/LR16IPT04, Institut Pasteur de Tunis, Université de Tunis El Manar, Tunis, Tunisia

**Keywords:** Cutaneous leishmaniasis, Apoptosis, *L. major*, *L. tropica*, MiRNA, Computational biology and bioinformatics, Parasitology

## Abstract

Leishmaniasis is a vector-borne infectious disease that affects many subtropical countries. *Leishmania (L.) major* and *L. tropica* are among the old-world causative agents and cause cutaneous leishmaniasis (CL). The parasite utilizes various mechanisms to evade host immune responses and facilitate intracellular survival. Among these mechanisms, apoptosis inhibition through altering the host cell miRNA expression profile plays a significant role in parasite survival and infection progression. Based on the in-silico analysis through the miRDB database and literature review, miR-4795-3p, miR-6785-5p, miR-5011-5p, and miR-155-5p were selected as miRNAs regulating apoptosis-related genes. The expression of selected miRNAs was evaluated in the skin biopsy lesions collected from *L. tropica* and *L. major*-infected patients by qRT-PCR. Our results showed that miR-155-5p, miR-5011-5p, and miR-6785-5p were significantly upregulated (*P* < 0.05) in *L. tropica-infected* patients. Similar expression patterns for miR-155-5p and miR-6785-5p, but with a higher magnitude, were found in the lesions of CL patients infected with *L. major*. Additionally, miR-4795-3p expression level was downregulated in this group. The KEGG pathway analysis indicated that these miRNAs target several pathways that play key roles during leishmaniasis. The results underscore that further investigation is needed to better understand the regulatory roles of these miRNAs in CL infection.

**Supplementary Information:**

The online version contains supplementary material available at 10.1038/s41598-025-03802-1.

## Introduction

 Leishmaniasis is a disease caused by an obligate intracellular parasite belonging to over 20 *Leishmania* species^1-3^. This vector-borne disease has different clinical manifestations, such as cutaneous, visceral, and mucocutaneous leishmaniasis, depending on the species involved, and among them, cutaneous leishmaniasis (CL) is the most prevalent form^4,5^. Leishmaniasis is transmitted by infected female sandflies from the *Phlebotomus* (Old World leishmaniasis) and *Lutzomyia* (New World leishmaniasis)^6^. This disease is characterized by painless lesions on exposed body areas^7^. CL is broadly noted in North Africa, the Americas, and the Near and Middle East^8^. Zoonotic CL, caused by *L. major*, and anthroponotic CL, caused by *L. tropica* are documented as the dominant forms of CL in the Middle East^9^.

The complex interactions between the *Leishman* parasite and the host’s immune system determined the outcome of CL. A balanced regulation of pro-inflammatory and anti-inflammatory signals governs this interaction^10^. *Leishmania* parasite utilizes various strategies to subvert host immune responses, and evade defense mechanisms, such as apoptosis, thereby enhancing its survival and proliferation^11,12^. It has been shown that the infection of bone marrow-derived macrophages (BMDMs) with *L. major* can facilitate a reduction in mitochondrial outer membrane permeabilization (MOMP), mitochondrial cytochrome c release, and caspase-3 activation. The alternation is suggested as a potential mechanism underlying delayed apoptosis in these cells^13^. In addition, *Leishmania* can regulate host cell apoptosis through interactions with members of the Bcl-2 family. The inhibition of apoptosis observed in monocyte-derived dendritic cells infected with *L. mexicana* is associated with an upregulation of the Bcl-xL protein^14^. It is also important to mention that the phosphoinositide 3-kinase (PI3 K)/Akt signaling pathway is crucial in inhibiting apoptosis by the *Leishmania* parasite^15,16^. MicroRNAs (miRNA), small non-coding RNAs with a length of 21 to 23 nucleotides, have also emerged as a potential regulator of apoptosis^17^. miRNAs are characterized by their ability to regulate gene expression at the post-transcriptional level^18,19^. They have an essential role in numerous human disease pathogeneses^20^. Various studies have shown that miRNAs may serve as a strategy for the *Leishmania* parasite to inhibit apoptosis^21-23^. Changes in miRNA expression level can occur in both the host and the parasite, and these alterations play a part in signaling manipulation of host cells and physiological processes, thus enabling the parasite to establish infection and escape the immune response^24^. As examples, integrated analysis of gene expression datasets followed by ex vivo validation showed that changes in the expression of miR-193b and miR-671, which was associated with the inflammatory response during localized CL caused by *L. braziliensis*, contributing to more effective treatment response and faster wound healing^25^. In another approach, the use of synthetic miRNA mimics of miR-340, in combination with miR-27a resulted in inhibition of of anti-inflammatory cytokines IL-10 and TGF-β1. These cytokines are upregulated during the infection of macrophages with *L. major*, leading to a decrease in parasite load^26^. Furthermore, elevated levels of miR-361-3p and miR-140-30 in the skin lesions caused by *L. braziliensis* infection were identified to be correlated with therapeutic failure using antimonial treatment and longer healing time for lesions, highlighting its potential to serve as a prognostic biomarker^27^. In addition, different investigations clearly demonstrate that several miRNAs undergo significant regulatory changes during an infection with *L. major*. Specifically, miR-210, miR-22, miR-155, and miR-133b are likely to be involved in the process of apoptosis^28^.

In the current study, we investigated the differential expression of four miRNAs, including miR-4795-3p, miR-6785-5p, miR-5011-5p, and miR-155-5p-predicted in silico to be linked to apoptosis in the skin lesions of CL patients infected with *L. major* and *L. tropica.* We also tried to provide a holistic overview of the regulatory role of the selected miRNA by assessing their diagnostic potential and other enriched pathways.

## Materials & methods

### Research design and bioinformatics analysis

In the first step, the miRDB database (http://mirdb.org) was implemented to mine the miRNA targeting CASP3, BCL2L1, and PIK3CG as apoptosis-related genes^29,30^. Result of this prediction were ranked by target score, filtering out any target with prediction score below 90%. MiRNAs gene that had the highest target score were selected as the final candidate for each mRNA target gene. Three candidate miRNAs were then evaluated for their comparative levels in the skin lesion samples of CL patients with quantitative RT-PCR. Further, miR-155-5p was included in our assessment based on a literature search^22,23,31-33^.

In the second step, to find other related target genes of the four selected miRNAs, we used the DIANA-miRPath v3.0 (https://dianalab.e-ce.uth.gr/html/mirpathv3) database, which incorporates data from three well-known tools for predicting miRNA target genes, including microT-CDS, TargetScan, and TarBase v7.0^34^. We set a threshold of 0.8 for the target score parameter in the database. Then, we adopted an integrated strategy to filter the predicted target genes for each miRNA from the above database. Unique and shared miRNA-target genes of four miRNA visualized using the Venny 2.1.0 tool (https://bioinfogp.cnb.csic.es/tools/venny/)^35^. KEGG (Kyoto Encyclopedia of Genes and Genomes) pathway enrichment analysis^36,37,38^ was performed to determine the linked pathways of four miRNAs. KEGG pathway enrichment analysis results were visualized using ggplot2 package.

## Sample collection and ethical statement

Skin lesion samples were collected from patients suspected of CL referred to Imam Reza Hospital and Ab-o Bargh Health Center in Mashhad, Iran, from January to October 2024. The inclusion criteria for including patients with CL in this study were as follows: having no prior anti-leishmanial treatment, presenting with an active lesion for up to one year (raised borders and without mucosal involvement), having positive diagnostic PCR results for either *L. tropica* or *L. major*, and aged between 15 and 75 years. Patients with systemic diseases, pregnancy, and diabetes were excluded from our study. Demographic information of the included patients, such as age and sex, along with the lesions’ characteristics, was recorded. Healthy control samples were obtained from volunteers undergoing blepharoplasty at Noor Eye Hospital in Tehran, Iran. The healthy volunteers participated in the study; none had a history of leishmaniasis. Non-invasive techniques were used to collect samples from skin lesions, which were then processed for two PCR tests to identify the species of *Leishmania*, as described by Taslimi et al.^39^.

In total, 17 CL patients, comprising ten infected with *L. tropica* and seven infected with *L. major*, and seven healthy individuals, were selected for this study. From each CL patient and healthy volunteer, one punch skin biopsies with a diameter of 2 millimeters were taken by a dermatologist. In the case of patients with multiple lesions, biopsies were taken from the edges of the active lesions with a duration of at least one month. The collected samples were immediately transferred to RNA Later solution (Qiagen GmbH, Hilden, Germany) and stored at −20 °C for later analysis. This investigation was performed in accordance with the guidelines and regulations of the Pasteur Institute of Iran. All participants were given information about the project, and written informed consent was obtained for the collection of clinical samples. All methods and consent forms were approved by the Research Ethics Committee of the Pasteur Institute of Iran (IR.PII.REC.1401.052).

## MiRNA extraction, cDNA synthesis, and quantitative real-time PCR

MiRNA was extracted from the skin biopsies using a miRNA extraction kit (Trans, China), following the manufacturer’s protocol. After that, one-step qRT-PCR was performed in an ABI Real-time PCR Detection System (Applied Biosystems, CA, USA), using 4X CAPITAL™ 1-Step qRT-PCR Green Master Mix (Biotechrabbit, Germany) as per the manufacturer’s protocol. The reaction volume was 10 µL, containing specific and universal primers (5 pmol). The thermal cycle was as mentioned: 50ºC for 5 min, 95ºC for 3 min, 40 cycles at 95ºC for 10 s, and 60ºC for 30 s. For One-step real-time RT-PCR, the primers were designed using the sRNAPrimerDB database (http://www.srnaprimerdb.com). The list of primers used in this study is presented in Table 1.

**Table 1:** List of primers utilized in qRT-PCR.

	Primer name	Sequence (5’- 3’)
miR-4795-3p	RP1 (specific)	GGACGGTAGCAAGCAAAGAGTGTGATCCAGAAGTG
	RP2 (specific)	GGGATTCTGGAAGATGATGATGACATATTATTAGC
miR-6785-5p	RP1 (specific)	GGACGGTAGCAAGCAAAGAGTGTGCACCATCATCC
	RP2 (specific)	GGGATTCTGGAAGATGATGATGACTGGGAGGGCGT
miR-5011-5p	RP1 (specific)	GGACGGTAGCAAGCAAAGAGTGTGGAGTGCATGGC
	RP2 (specific)	GGGATTCTGGAAGATGATGATGACTATATATACAG
miR-155-5p	RP1 (specific)	GGACGGTAGCAAGCAAAGAGTGTGAACCCCTATCA
	RP2 (specific)	GGGATTCTGGAAGATGATGATGACTTAATGCTAAT
SNORD46	SNORD46-F	AGAATCCTTAGGCGTGGTTGT
	SNORD46-R	ATGACAAGTCCTTGCATTGGC
Universal Primers	P1 (universal)	GGACGGTAGCAAGCAAAGAGTGTG
	P2 (universal)	GGGATTCTGGAAGATGATGATGAC

## Statistical methods

The data analysis for this study was conducted using R (version 4.4.2, https://www.r-project.org/)^40^. The analysis encompassed data processing, normalization, statistical testing, and visualization of fold changes (FC) for four miRNAs (miR-155-5p, miR-5011-5p, miR-4795-3p, and miR-6785-5p) across three groups: Healthy individuals, *L. major*-infected patients, and *L. tropica*-infected patients. FC for each miRNA was calculated using the delta-delta Ct method, normalized to snoRNA, C/D Box 46 (SNORD46)^25^ as the reference gene. Log2 transformation was applied to the fold changes to normalize the data for subsequent analyses. Descriptive statistics, including measures such as median and interquartile range (IQR), were used to summarize fold-change distributions across the groups. To compare miRNA expression between the groups, the Kruskal-Wallis test was employed, followed by pairwise comparisons using Dunn’s test with Holm’s method for adjusting *p*-values to control for multiple testing.

The diagnostic potential of individual miRNA to differentiate between groups was assessed using receiver operating characteristic (ROC) analysis. Metrics such as the area under the curve (AUC), sensitivity, specificity, and accuracy were calculated. ROC analyses were summarized for comparisons between healthy vs. *L. major*-infected patients, healthy vs. *L. tropica*-infected patients, and *L. major* vs. *L. tropica*-infected patients. In addition, the combiROC R package (Ferrari et al., 2022) was used to evaluate the combined diagnostic potential of the four microRNAs as a biomarker panel^41^. This package applies binomial logistic regression to model the probability of binary outcomes, such as group membership, based on the miRNA expression data. The resulting model improves diagnostic accuracy while providing insights into the contribution of each miRNA to the combined prediction. Finally, pairwise correlations between miRNAs were examined using Spearman’s rank correlation coefficient, and scatterplots with linear regression fits were generated to illustrate these relationships across all samples and stratified by group. Statistical significance was set at *P* < 0.05.

## Results

### Demographic and clinical characteristics of ***L. major***-and ***L. tropica***-infected patients and healthy volunteers

As depicted in Table 2, this study included 10 *L. tropica*-infected CL patients and 7 *L. major*-infected CL patients with a mean age of 40.30 ± 20.76 and 41.14 ± 20.73 years old, respectively. Four (40%) of *L. tropica* patients were females, and six (60%) were males. Meanwhile, one (14%) of *L. major* patients were female, and six (86%) were male. Seven healthy volunteers were included, with a mean age of 54 ± 12.66 years old, including six (86%) females and one (14%) male. Two (20%) *L. tropica*-infected patients had a single lesion. The rest had more than one lesion. Four (57%) *L. major*-infected patients had only one lesion, and others had more lesions. The average disease duration and lesion size in the patients infected with *L. tropica* and *L. major* were 3.6 ± 2.3 and 2.5 ± 1.4 months, 3.15 ± 1.97 and 31.57 ± 40.27 cm^2^, respectively. Further, we identified no significant differences in age, size, number, and duration of lesions between patients caused by both species (Table 1). All individual values are shown in Supplementary File S1.

**Table 2:** Detailed demographic and clinical characteristics of the study participants.

Characteristics		Healthy	*L. major*	*L. tropica*	*P*-value^1^
**Sex**	Male	1 (14%)	6 (86%)	6 (60%)	0.029
	Female	6 (86%)	1 (14%)	4 (40%)	
**Age**	Median (Q1-Q3)	54.00 (41.00–66.00)	41.00 (19.00–60.00)	29.50 (24.00–64.00)	0.3
	Mean years	54 ± 12.66	41.14 ± 20.73	40.30 ± 20.76	
**Lesion**	Duration Median (Q1-Q3) (month)	-	3.00 (1.00–3.50)	4.00 (1.00–5.00)	0.3
	Duration Mean ± SD (month)	-	2.5 ± 1.4	3.6 ± 2.3	
	Number = 1	-	4 (57%)	2 (20%)	0.2
	≥ 2	-	3 (43%)	8 (80%)	
	Size of punch biopsy (cm2) Median (Q1-Q3)	-	17.50 (1.00–70.00)	2.50 (2.00–4.00)	0.2
	Size of punch biopsy (cm2) Mean (SD)	-	31.57 (40.27)	3.15 (1.97)	

SD: standard deviation.

^1^Fisher’s exact test; Kruskal-Wallis rank sum test.

#### Selection of candidate miRNAs and differential expression analysis

Results of mining the potential miRNAs target genes by the miRDB database highlighted that miR-4795-3p, miR-6785-5p, and miR-5011-5p were among the targets with the highest target score and lowest target rank. Additionally, miR-155-5p was chosen based on a literature review of various studies^22,23,31-33^.

**Fig. 1:**
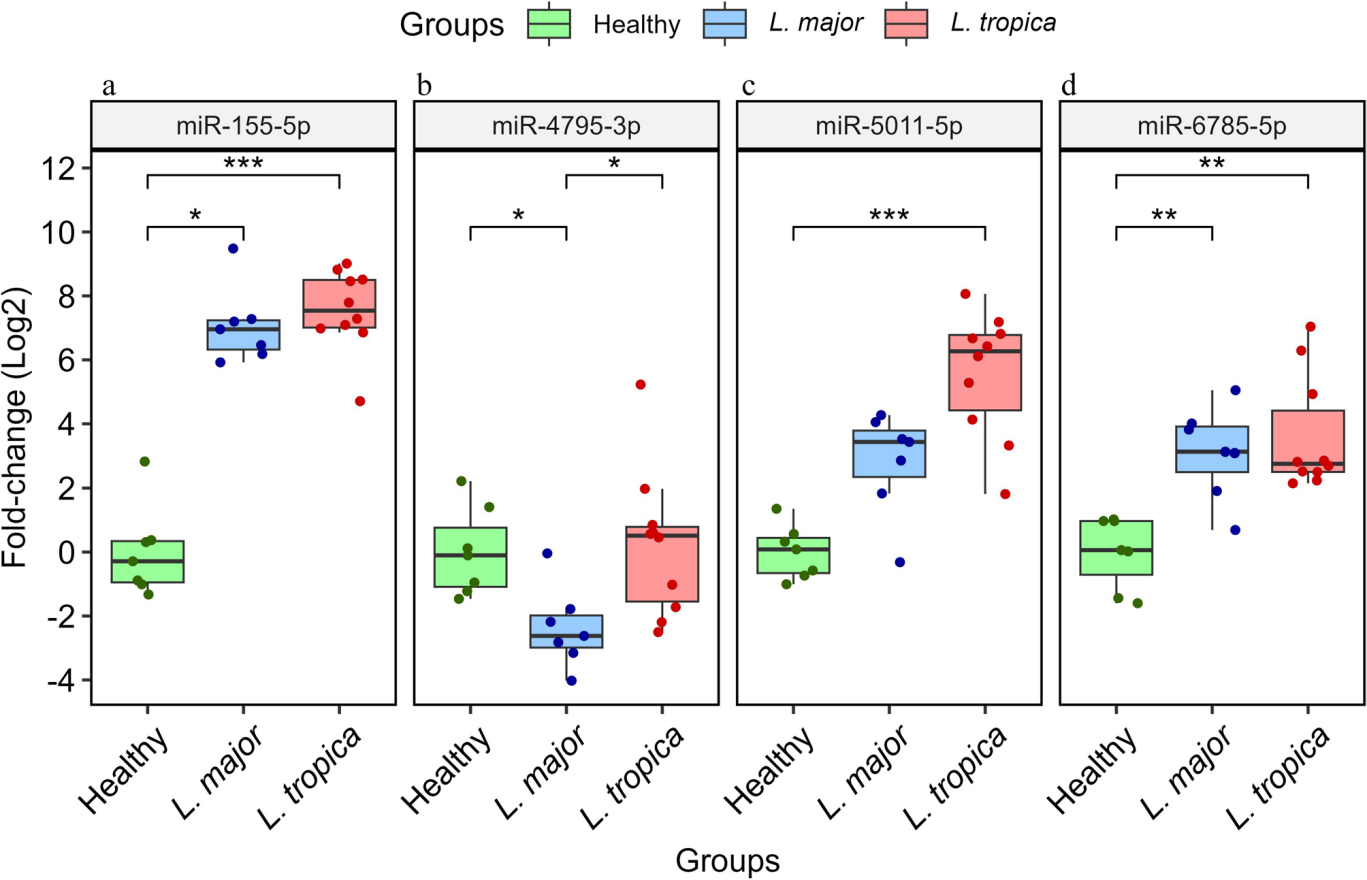
The expression levels of four candidate miRNAs between *L. major*- and *L. tropica*-infected patients and healthy group. (**a**) miR-155-5p, (**b**) miR-4795-3p, (**c**) miR-5011-5p, (**d**) miR-6785-5p. Data are normalized using SNORD46 as the control gene. *P* < 0.05 is considered statistically significant. The results were shown as the median (Q1-Q3).

Based on the magnitude of fold changes of the selected miRNA genes, all miRNAs except for miR-4795-3p were significantly up-regulated in the skin lesion of *L. tropica*-infected patients compared to the healthy group (*P* < 0.05) (Fig. 1). In the *L. major*-infected group, expression of miR-155-5p and miR-6785-5p (*P* < 0.05) were increased, while miR-4795-3p demonstrated a notable downregulation (*P* < 0.05). When comparing *L. major*-infected patients to those infected with *L. tropica*, only miR-4795-3p exhibited a significant decrease in expression (*P* < 0.05). Pair-wise comparison data are summarized in Table 3.

**Table 3:** Summary of pairwise comparison for all candidate miRNAs.

Between-Group Comparisons Significance testing results with adjusted *p*-values				
miRNA	**Groups**	**Adjusted** ***P*-value**	**Adjusted Significance**	
miR-155-5p	Healthy vs. *L. major*	0.0130	*	
	Healthy vs. *L. tropica*	0.0005	***	
	*L. major* vs. *L. tropica*	0.4030	ns	
miR-4795-3p	Healthy vs. *L. major*	0.0203	*	
	Healthy vs. *L. tropica*	0.9477	ns	
	*L. major* vs. *L. tropica*	0.0195	*	
miR-5011-5p	Healthy vs. *L. major*	0.0987	ns	
	Healthy vs. *L. tropica*	0.0002	***	
	*L. major* vs. *L. tropica*	0.0987	ns	
miR-6785-5p	Healthy vs. *L. major*	0.0056	**	
	Healthy vs. *L. tropica*	0.0030	**	
	*L. major* vs. *L. tropica*	0.9575	ns	

## Differential expression levels and diagnostic performance for the candidate miRNAs between the studied groups

**Fig. 2:**
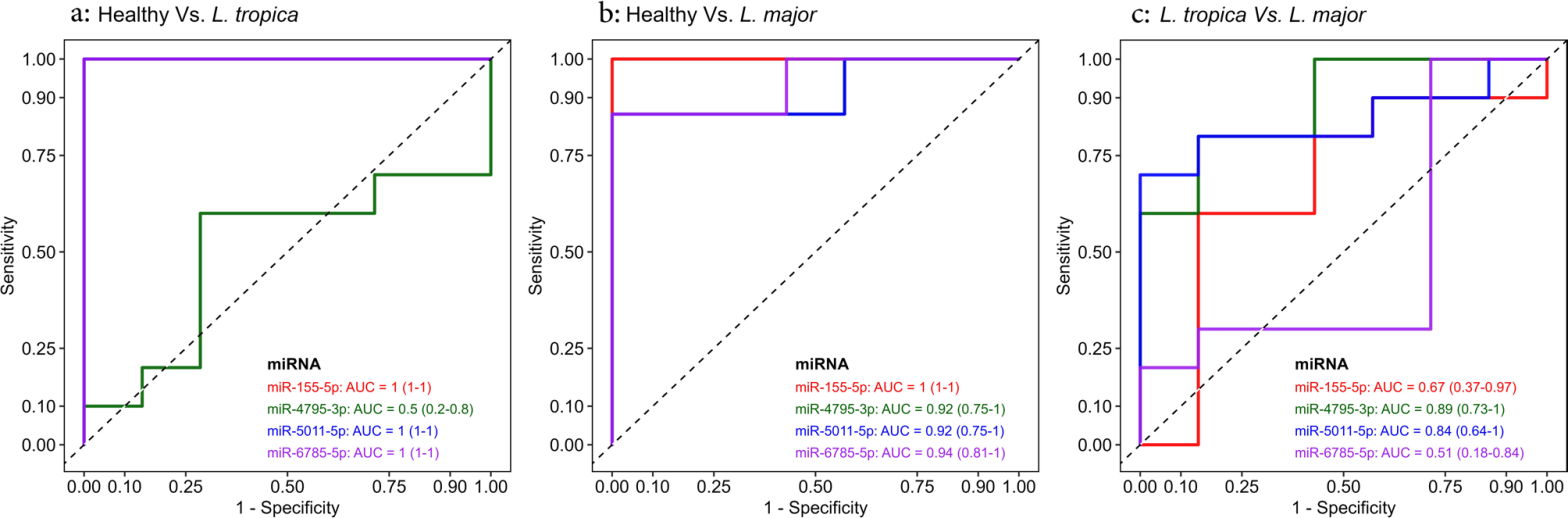
Receiver operating characteristic (ROC) analysis of four candidate miRNAs. (**a**) Results showed significant diagnostic values for all candidate miRNAs except for miR-4795-3p in *L. tropica*-infected patients and the healthy controls. (**b**) The analysis indicated significant diagnostic values for all candidate miRNAs between *L. major*-infected patients and the controls. (**c**) Results showed significant diagnostic values for miR-4795-3p and miR-5011-5p between *L. major* and *L. tropica*-infected patients.

The diagnostic power of candidate miRNAs was evaluated using ROC and binomial logistic regression on the qRT-PCR data, and the AUC of ROC was measured for each candidate (*P* < 0.05). As depicted in Fig. 2a, three candidate miRNAs, including miR-155-5p, miR-5011-5p, and miR-6785-5p, significantly discriminated *L. tropica*-infected patients from healthy controls (AUC = 1.00, *P* < 0.05), with 100% sensitivity, specificity, and accuracy at cut-off values of 16.613, 3.034, and 3.233, respectively. Additionally, in *L. major*-infected patients, miR-155-5p was also significantly distinguished between the groups (AUC = 1.00, *P* < 0.05), with 100% sensitivity, specificity, and accuracy at a cut-off value of 33.863 (Fig. 2b). Findings for the other three miRNAs, including miR-4795-3p, miR-5011-5p, and miR-6785-5p, were also promising in the case of comparison between *L. major*-infected patients and healthy subjects, with AUCs of 0.92, 0.92, and 0.94 and *P* < 0.05, respectively. They showed 86% sensitivity, 100% specificity, and 93% accuracy at the cut-off points of 0.327, 3.058, and 2.893, respectively (Fig. 2b). Moreover, using ROC, we evaluated the discriminating power of candidate miRNAs between the two groups of CL patients. In this case, miR-4795-3p and miR-5011-5p distinguish between the two groups significantly (AUC = 0.89, 0.84, *P* < 0.05, respectively) with 80% and 70% sensitivity, 86% and 100% specificity, 82% accuracy at the cut-off value of 0.297 and 29.131 (Fig. 2c). Summary of ROC analysis data are presented in Table 4.

**Table 4:** Summary of ROC analysis data. Optimal cutoff value, specificity, and sensitivity of four miRNAs for diagnosis of *L. major* and *L. tropica*-infected patients.

miRNA Performance in Classifying Groups Evaluation of miRNA markers across different group comparisons											
Groups	miRNA	Cutoff	AUC	ACC	SEN	SPE	PPV	NPV	PLR	NLR	*P*-value
Healthy vs. *L. major*	miR-155-5p	33.863	100	100	100	100	100	100	Inf	0.000	0.000
Healthy vs. *L. major*	miR-4795-3p	0.327	92	93	86	100	100	88	Inf	0.143	0.003
Healthy vs. *L. major*	miR-5011-5p	3.058	92	93	86	100	100	88	Inf	0.143	0.003
Healthy vs. *L. major*	miR-6785-5p	2.893	94	93	86	100	100	88	Inf	0.143	0.004
Healthy vs. *L. tropica*	miR-155-5p	16.613	100	100	100	100	100	100	Inf	0.000	0.000
Healthy vs. *L. tropica*	miR-4795-3p	1.229	50	65	60	71	75	56	2.100	0.560	0.519
Healthy vs. *L. tropica*	miR-5011-5p	3.034	100	100	100	100	100	100	Inf	0.000	0.000
Healthy vs. *L. tropica*	miR-6785-5p	3.233	100	100	100	100	100	100	Inf	0.000	0.000
*L. major* vs. *L. tropica*	miR-155-5p	155.572	67	71	60	86	86	60	4.200	0.467	0.885
*L. major* vs. *L. tropica*	miR-4795-3p	0.297	89	82	80	86	89	75	5.600	0.223	0.003
*L. major* vs. *L. tropica*	miR-5011-5p	29.131	84	82	70	100	100	70	Inf	0.300	0.009
*L. major* vs. *L. tropica*	miR-6785-5p	7.888	51	71	70	71	78	62	2.450	0.420	0.481

**Fig. 3:**
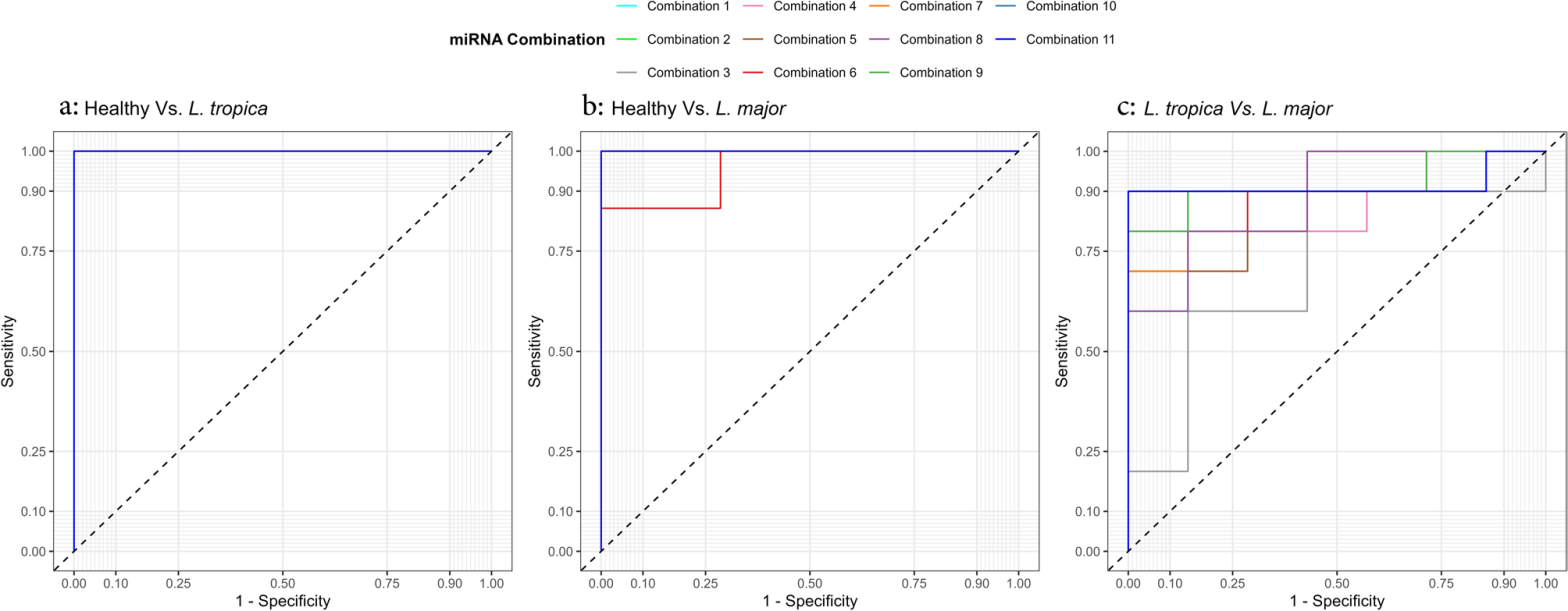
The ROC curve analysis is used to assess all combination models of miRNAs. (**a**) Healthy vs. *L. tropica*, (**b**) Healthy vs. *L. major*, (**c**) *L. tropica* vs. *L. major*.

Our analysis demonstrated that the candidate miRNAs have high diagnostic capabilities. We assert that combining our four miRNAs significantly enhances diagnostic efficacy in distinguishing between CL patients and healthy individuals and differentiating CL patients based on their causative species, specifically *L. tropica* and *L. major*. Figure 3a and b, and Table 5 confirm that all models developed to distinguish CL patients from healthy samples achieved remarkably high specificity (over 90%). This unequivocally indicates that all combination models effectively and accurately identify CL patients. In addition, we found that except for three combination models, 1, 3, and 8, the other combination models had high specificity (100%), and 5 of these combinations had high diagnostic values (≥ 90) to discriminate between *L. tropica*-infected patients and *L. major*-infected patients which are shown in Fig. 3c; Table 5.

**Table 5:** Specificity and sensitivity of all combined models of four miRNAs in healthy vs. *L. tropica*, healthy vs. *L. major*, and *L. tropica* vs. *L. major.*

Number	miRNA combination	AUC	Sensitivity	Specificity	Accuracy	miRNA combination	AUC	Sensitivity	Specificity	Accuracy
	Healthy vs. *L. tropica*					Healthy vs. *L. major*				
**1**	miR-155-5p + miR-4795-3p	100	100	100	100	miR-155-5p + miR-4795-3p	100	100	100	100
**2**	miR-155-5p + miR-5011-5p	100	100	100	100	miR-155-5p + miR-5011-5p	100	100	100	100
**3**	miR-155-5p + miR-6785-5p	100	100	100	100	miR-155-5p + miR-6785-5p	100	100	100	100
**4**	miR-4795-3p + miR-5011-5p	100	100	100	100	miR-4795-3p + miR-5011-5p	100	100	100	100
**5**	miR-4795-3p + miR-6785-5p	100	100	100	100	miR-4795-3p + miR-6785-5p	100	100	100	100
**6**	miR-5011-5p + miR-6785-5p	100	100	100	100	miR-5011-5p + miR-6785-5p	96	86	100	93
**7**	miR-155-5p + miR-4795-3p + miR-5011-5p	100	100	100	100	miR-155-5p + miR-4795-3p + miR-5011-5p	100	100	100	100
**8**	miR-155-5p + miR-4795-3p + miR-6785-5p	100	100	100	100	miR-155-5p + miR-4795-3p + miR-6785-5p	100	100	100	100
**9**	miR-155-5p + miR-5011-5p + miR-6785-5p	100	100	100	100	miR-155-5p + miR-5011-5p + miR-6785-5p	100	100	100	100
**10**	miR-4795-3p + miR-5011-5p + miR-6785-5p	100	100	100	100	miR-4795-3p + miR-5011-5p + miR-6785-5p	100	100	100	100
**11**	miR-155-5p + miR-4795-3p + miR-5011-5p + miR-6785-5p	100	100	100	100	miR-155-5p + miR-4795-3p + miR-5011-5p + miR-6785-5p	100	100	100	100
***L. tropica vs. L. major***										
**1**	miR-155-5p + miR-4795-3p	91	90	86	88	miR-155-5p + miR-4795-3p + miR-5011-5p	87	70	100	82
**2**	miR-155-5p + miR-5011-5p	84	70	100	82	miR-155-5p + miR-4795-3p + miR-6785-5p	89	80	86	82
**3**	miR-155-5p + miR-6785-5p	71	90	57	76	miR-155-5p + miR-5011-5p + miR-6785-5p	91	80	100	88
**4**	miR-4795-3p + miR-5011-5p	86	70	100	82	miR-4795-3p + miR-5011-5p + miR-6785-5p	91	90	100	94
**5**	miR-4795-3p + miR-6785-5p	89	70	100	82	miR-155-5p + miR-4795-3p + miR-5011-5p + miR-6785-5p	91	90	100	94
**6**	miR-5011-5p + miR-6785-5p	90	80	100	88					

**Fig. 4:**
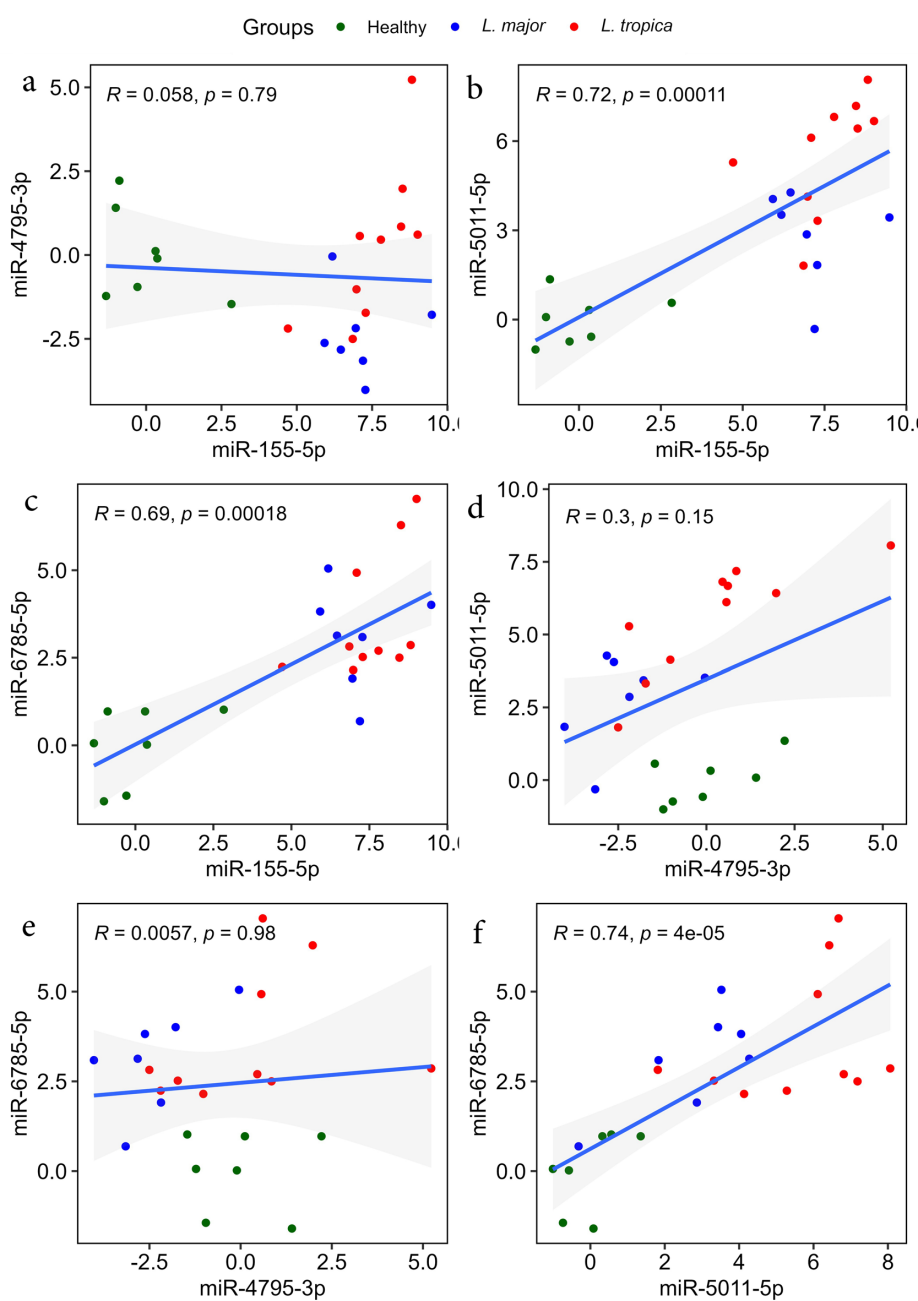
Scatter plots of Pearson correlation analysis between miRNA pairs. Plots show the relationships between miRNAs expression levels in total. Results show statistically significant relationships between three miRNA pairs. (**a**) miR-4795-3p and miR-155-5p, (**b**) miR-5011-5p and miR-155-5p, (**c**) miR-6785-5p and miR-155-5p, (**d**) miR-5011-5p and miR-4795-3p, (**e**) miR-6785-5p and miR-4795-3p, (**F**) miR-6785-5p and miR-5011-5p.

The pair-wise correlation of four miRNAs between CL patients and healthy individuals showed several statistically significant positive correlations in pair-wise miRNAs. Specifically, the expression of miR-6785-5p showed a positive correlation with miR-5011-5p expression (*r* = 0.74, *P* < 0.05) (Fig. 4f). In addition, this analysis demonstrates a statistically significant positive correlation between the expression of miR-5011-5p and miR-155-5p (*r* = 0.72, *P* < 0.05) (Fig. 4b). Furthermore, our analysis highlighted a positive correlation between miR-6785-5p and miR-155-5p (*r* = 0.69, *P* < 0.05) (Fig. 4c). Notably, the values for all pair-wise correlations are shown in Fig. 4.

**Fig. 5:**
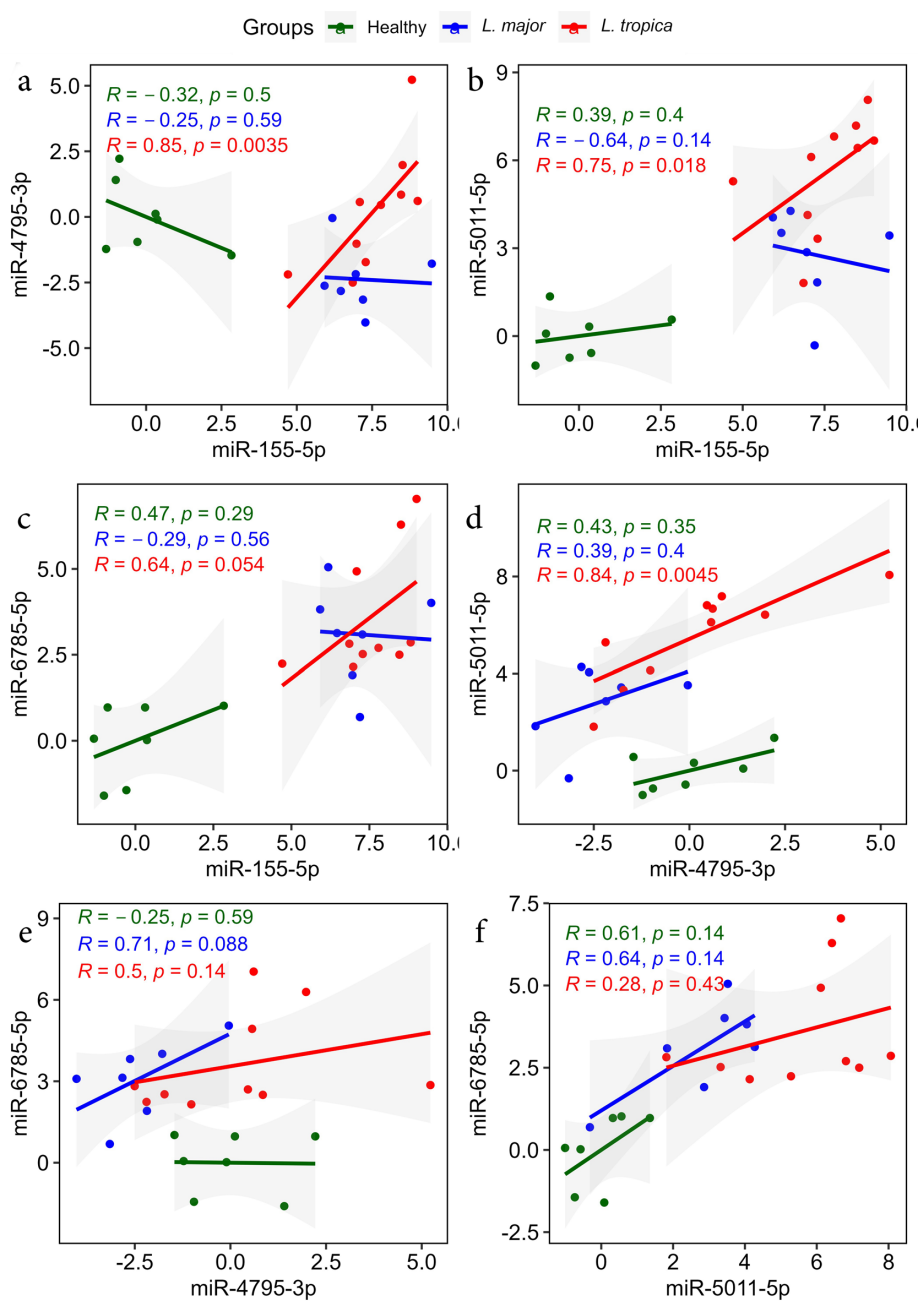
Scatter plots of Pearson correlation analysis between miRNA pairs. Plots show the relationships between miRNAs expression levels by group. Results show statistically significant relationships between three miRNA pairs in *L. tropica*-infected patients. (**a**) miR-4795-3p and miR-155-5p, (**b**) miR-5011-5p and miR-155-5p, (**c**) miR-6785-5p and miR-155-5p, (**d**) miR-5011-5p and miR-4795-3p, (**e**) miR-6785-5p and miR-4795-3p, (**F**) miR-6785-5p and miR-5011-5p.

Another Pearson’s correlation analysis was done to determine the pair-wise correlation in groups including *L. tropica*-infected patients, *L. major*-infected patients, and healthy controls. This analysis demonstrated significantly positive correlations in pair-wise miRNAs in *L. tropica*-infected patients. They included the highest significant correlation between miR-4795-3p and miR-155-5p (*r* = 0.85, *P* < 0.05) (Fig. 5a), which is followed by another positive correlation between miR-5011-5p and miR-4795-3p (*r* = 0.84, *P* < 0.05) (Fig. 5d). Moreover, another positive correlation was determined between miR-5011-5p and miR-155-5p (*r* = 0.75, *P* < 0.05) (Fig. 5b). The values of this analysis are shown in Fig. 5.

## KEGG pathway enrichment of the candidate miRNA

**Fig. 6:**
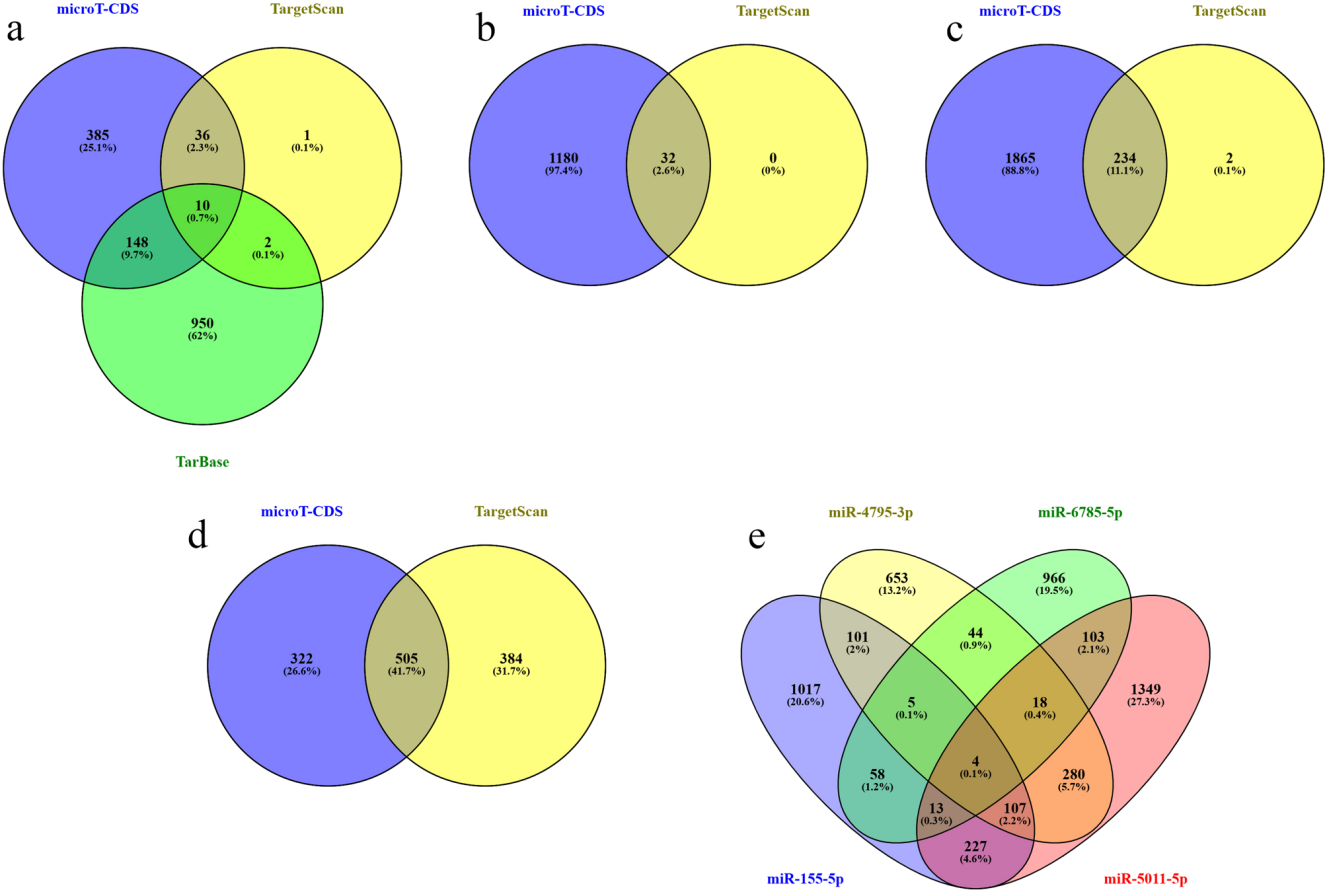
Venn diagram for target genes of miRNAs. (**a**) miR-155-5p (**b**) miR-4795-3p (**c**) miR-5011-5p (**d**) miR-6785-5p (**e**) the overlaps target genes of four miRNAs.

To predict the other target genes of miR-155-5p, miR-4795-3p, miR-5011-5p, and miR-6785-5pA, DIANA-miRPath v3.0 was used. As depicted in Fig. 6 (a-e), different numbers of potential target genes of each selected miRNA were observed. A total of 1532, 1212, 2101, 1211 non-redundant targets genes were predicted for miR-155-5p, miR-4795-3p, miR-5011-5p, and miR-6785-5p, respectively.

**Fig. 7:**
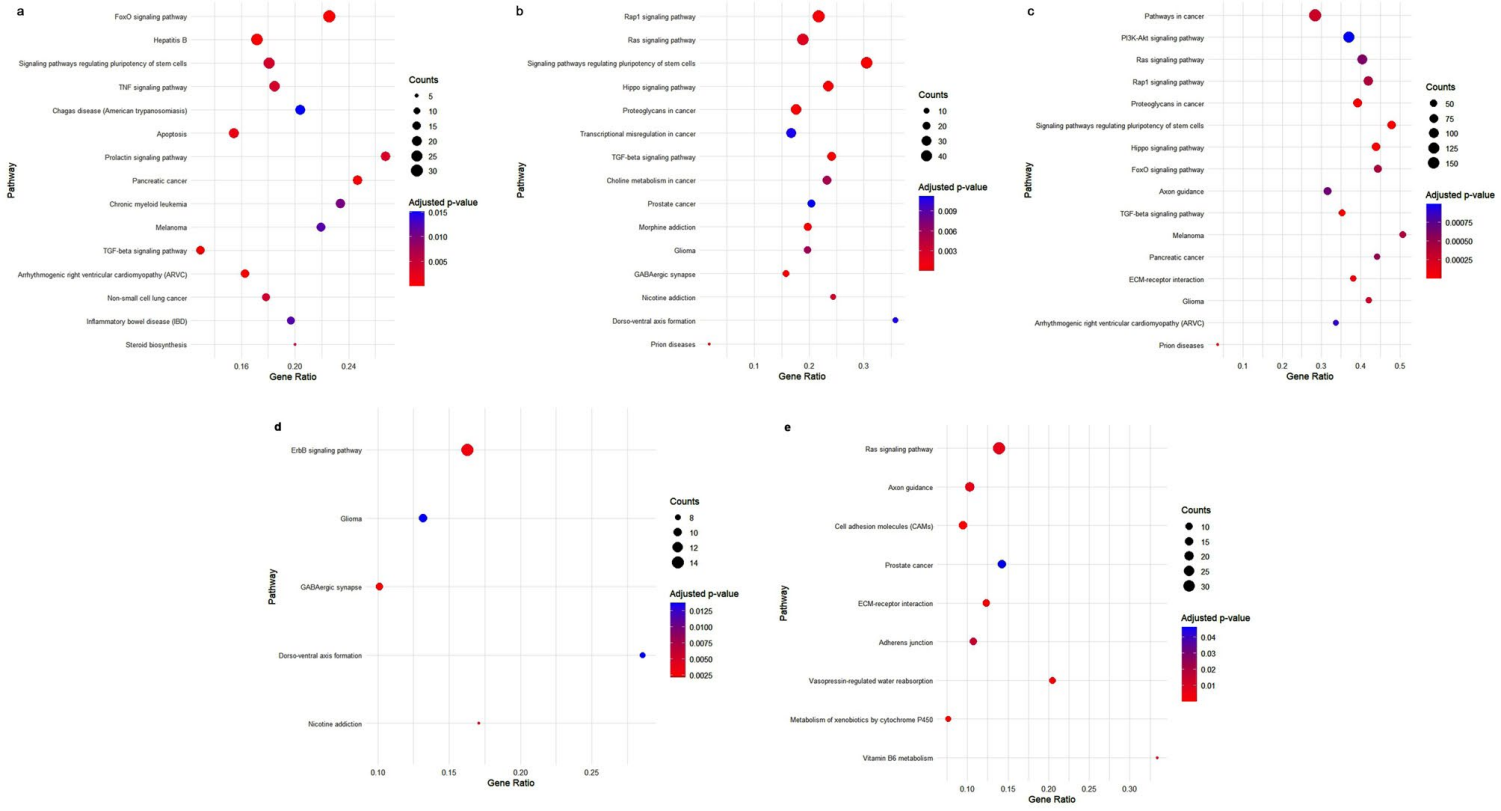
KEGG pathway enrichment analysis of miRNAs. (**a**) miR-155-5p (**b**) miR-5011-5p (**c**) signaling pathways targeted by four miRNAs (**d**) miR-4795-3p (**e**) miR-6785-5p.

Functional enrichment analysis of predicted miRNA targets showed that the top enriched pathways by miR-155-5p (Fig. 7a) were Hepatitis B, Arrhythmogenic right ventricular cardiomyopathy (ARVC), TGF-beta signaling pathway, and FoxO signaling pathway while Prion diseases, Signaling pathways regulating pluripotency of stem cells, TGF-beta signaling pathway, and Hippo signaling pathway were among the highest enriched pathways by miR-5011-5p (Fig. 7b). Genes targeted by miR-4795-3p (Fig. 7d) were mainly enriched in GABAergic synapse, Nicotine addiction, ErbB signaling pathway, and Dorso-ventral axis formation. Regarding miR-6785-5p, Metabolism of xenobiotics by cytochrome P450, ECM-receptor interaction, Cell adhesion molecules (CAMs), and Vasopressin-regulated water reabsorption were mainly induced (Fig. 7e). The enriched KEGG pathways of miRNA genes targeted by each selected miRNA are presented in Fig. 7(a-e) (*P* < 0.05).

## Discussion

Protozoan parasites, including *Leishmania*, induce the miRNA-mediated post-transcriptional regulation of genes involved in different cellular pathways, such as the inflammatory response and apoptosis, to enhance their survival and pathogenicity in infected host cells^21^. Various studies have shown that miRNAs may serve as a strategy for the *Leishmania* parasite to inhibit apoptosis^11,42^; however, the expression pattern of apoptosis-associated miRNA in the context of leishmaniasis caused by different species has not been investigated more. Accordingly, in the current study, we evaluated the local expression of four candidate miRNAs predicted to be linked to apoptosis in the skin lesions of CL patients caused by *L. major* and *L. tropica*, and healthy individuals using the qRT-PCR method.

Our analysis identified distinct expression levels of miR-155-5p, miR-6785-5p, miR-5011-5p, and miR-4795-3p, in the skin lesions of patients infected with *L. major* and *L. tropica*. MiR-155 is recognized to have a potential role in regulating inflammation, immune response, several cancer-related pathways^32^. In the context of leishmaniasis, it is identified to be differentially regulating the immune responses and outcome of different forms of leishmaniasis^43^. For instance, it is significantly expressed in monocyte-derived macrophages infected by *L. braziliensis* and *L. major*, which might be linked to modulating the inflammatory network^44,45^. Regarding its potential involvement in apoptosis during leishmaniasis, Lago et al. showed a strong inverse correlation between the expression of Annexin V and miR-155-5p in macrophages infected with *L. braziliensis*. They suggested that miR-155-5p may be involved in the apoptosis regulation in the infected macrophages^22^. In another report, using miRNA-155 inhibitor and miRNA-15a mimic in macrophages infected by *L. major* revealed that the number of apoptotic cells were increased. Further, in the case of miR-155 inhibitor, they also noticed caspase-3/7 overexpression and a decrease in parasite load^46^. Similarly, we herein could identify overexpression of miR-155-5p in CL lesions caused by *L. major* and *L. tropica* and speculated that it may have a role in modulating apoptotic pathways and promoting parasite persistence.

Regarding miR-6785-5p, it has been studied in various contexts, such as psoriasis, gastric cancer, colorectal cancer, and type 2 diabetes mellitus^47-53^. Further, upregulated miR-6785-5p was predicted to regulate the activation and differentiation of Th1 and Th17 cells^48^. In gastric cancer, it is found to be involved in suppressing tumor growth by inhibiting BCL2^49^. However, the role of this miRNA in *Leishmania* infection is still not characterized. In our current study, based on the expression pattern of this miRNA in the lesions of *L. tropica* and *L. major-*infected patients, and regarding the central role of its target gene (BCL2L1) in the apoptosis pathway, it can be reasonable to consider that miR-6785-5p may have a pathogenic role during the infection and increase parasite survival. Further, we could show that the expression of miR-6785-5p and miR-155-5p had a statistically significant positive correlation (*r* = 0.7), suggesting a potential functional relationship between these two miRNAs. However, understanding the detailed mechanism involved in this regulatory network needs further investigation. ROC curve analysis for combination models of these two miRNAs in *L. tropica- and L. major-*infected patients also revealed significant diagnostic potential. This finding demonstrates the potential of these miRNAs to be evaluated as biomarkers for the detection and diagnosis of acute CL. Therefore, further research is inevitable to validate the role of the upregulated miRNA in apoptosis in a larger cohort, as this could pave the way for their use in monitoring infection progression and therapeutic purposes in CL disease. MiR-4795-3p and miR-5011-5p were other studied miRNA genes. While deregulation of miR-4795-3p has been documented in myocardial infarction^54^ and endometrial endometrioid Cancer^55^, its role in leishmaniasis remains unexplored. Notably, the underlying mechanisms of miR-4795-3p affecting intracellular processes are not fully understood, highlighting the need for further study. MiR-5011-5p is known to be associated with colorectal cancer, polycystic ovary syndrome, cervical dystonia, multiple myeloma, asymptomatic monoclonal gammopathy of undetermined significance, gliomas, glioblastoma, and CL caused by *L. braziliensis*^56-62^. It has been shown that miR-5011-5p may target SMAD6 and SMAD7 genes in the TGFβ signaling pathway. Additionally, targeting *wnt3a* and *lrp6* genes through the WNT signaling pathway has been predicted^56^. A recent report showed that target genes of miR-5011-5p were involved in regulating the pluripotency of stem cells, the TGFβ signaling pathway, proteoglycans in cancer, and the Rap1 signaling pathway. MAPK3 was the common target gene in these enriched pathways^57^. In the case of CL due to *L. braziliensis*, the elevated level of miR-5011-5p expression was noted, and CXCL9, CXCL10, GBP5, and IDO1 were introduced as miRNA’s possible target genes^62^. Our result was consistent with these findings in the case of miR-5011-5p expression pattern in CL caused by *L. tropica*. Overexpression of three out of four predicted miRNA-related apoptosis in *L. tropica* skin lesions may be present a strategy to modulate their possible target genes in the lesions and contribute to the specific characteristics of lesions compared to *L. major*.

Since miRNA can target multiple genes^63^, we further investigate to predict potential downstream target genes and the pathways in which miRNAs mentioned above could be involved.

The KEGG pathway enrichment analysis indicated that miR-155-5p can target several pathways involved in *Leishmania*-infection progression. These pathways include the TNF signaling pathway, FOXO signaling pathway, Apoptosis, and TGF-β signaling pathway. TNF is shown to play an essential role in leishmaniasis pathogenesis and the outcome of the infection^64-67^. FOXO transcription factors and proteins are known to have a role in cell death, survival, and apoptosis. It is also known that it plays a pivotal role in regulating the immune and inflammatory response of the human body against various infections^68,69,70^. Roy et al. (2019) have shown that *Leishmania* can utilize the SIRT1/FOXO1 axis to affect PD-1 signaling, which results in intracellular parasite survival^71^. TGF-β can reduce pathogen burdens and tissue injuries in some cases of infection^72^. It is shown that TGF-β has a dual role in the *Leishmania* infection, which is important for the wound healing process and parasite survival through immune suppression activity by TGF-β^73^. These findings collectively underscore the role played by miR-155-5p during CL and the need for further investigation into this miRNA as a potential target for managing the infection.

The enriched pathways by miR-6785-5p included several pathways, like Ras signaling and cell adhesion molecules (CAMs). CAM are membrane-associated cell surface glycoproteins playing important roles in cell recognition, adhesion, migration, and differentiation^74^. The ability of cells to adhere through specific molecular interactions plays a critical role in a wide array of biological processes, including hemostasis, the immune response, inflammation, embryogenesis, and the development of neuronal tissue^75^. Pinheiro et al.. (2006) showed that *Leishmania* infection inhibited the adhesion of macrophages to connective tissue *via* the impairment of function in β−1 integrin^76^. The result of enrichment analysis can be another reason for considering a pathogenic role for miR-6785-5p in CL disease. Further research is needed to fully understand the mechanism of action.

The pathway enrichment analysis of miR-4795-3p showed that this miRNA is involved in several pathways, including the ErbB signaling pathway, demonstrating relevance to leishmaniasis. The ErbB family of receptor tyrosine kinases comprises ErbB1-ErbB4. ErbB1 is more commonly known as the epidermal growth factor (EGF) receptor, and these molecules are also referred to as the EGFR and HER2- HER4^77^. The EGF system is present in various human organs and plays a significant role in cell proliferation, differentiation, migration, and apoptosis during embryogenesis and postnatal development^78^. It has been shown that during *Leishmania* infection, genes involved in the ErbB signaling pathway were upregulated, which can affect macrophage polarization and the expression of pro-survival signals, favoring the parasite^79^. The downregulated level of this miRNA in *L. major*-infected patients in our study may represent a compensatory mechanism by the host to mitigate the parasite-induced dysregulation.

We could also document the enrichment of the Ras signaling pathway and TGF-β signaling pathway among the main pathways induced by miR-5011-5p. Ras is a GTP-binding protein that plays multiple important roles in cell activation, including proliferative and inflammatory responses^80^. Ras is recognized to have a key role in the progression of *Leishmania* infection, as it is understood that the *Leishmania* parasite can manipulate host Ras isoforms to promote anti-inflammatory responses of the host. Furthermore, it is well-known that this signaling pathway is essential for parasite internalization into host cells^81,82^. The overexpression of this miRNA in our studied *L. tropica*-infected patients may reflect a strategic immune modulation used by the pathogen to ensure its survival.

Moreover, the KEGG pathway enrichment analysis for all studied miRNAs showed that the targeted genes were involved in several pathways, including the PI3 K-Akt signaling pathway, the Ras signaling pathway, the TGF-β signaling pathway, and the FOXO signaling pathway, which also have a pivotal role in the leishmaniasis pathogenesis. Clearly, miRNAs are emerging as a helpful tool that provides several benefits, including their presence in serum samples as a non-invasive detection and their capacity to be used as a diagnostic biomarker or treatment option.

However, this study has limitations, including a small sample size, clinical heterogeneity, strict criteria to predict and include possible miRNA, which could induce selection bias. Further research is required to validate the observed expression pattern and biomarker potential in a larger cohort, as well as to validate the candidate miRNAs’ functions in apoptosis in CL disease.

In conclusion, we highlighted the deregulation of four potential miRNAs linked to apoptosis in the skin lesions of CL due to *L. major and L. tropica.* Further, ROC curve analysis for combination models of the candidate miRNA revealed significant diagnostic potential in the acute stage of CL disease. However, further study is warranted to validate the clinical and functional significance of the selected miRNA in CL. Such a study might lay a foundation for introducing new targets for effective diagnosis or therapeutic purposes in CL disease.

## Electronic supplementary material

Below is the link to the electronic supplementary material.

Supplementary Material 1

## Data Availability

The authors confirm that the data supporting the findings of this study are available within the article and/or its supplementary file.
