# Supplementary material for: Altered miRNA expression in the lesions of cutaneous leishmaniasis caused by L. major and L. tropica with insights into apoptosis regulation
Source: Sci Rep. 2025 Jul 1;15:20680. doi: 10.1038/s41598-025-03802-1 (PMC12219827; doi:10.1038/s41598-025-03802-1)

Supplementary Information

Altered miRNA expression in the lesions of cutaneous leishmaniasis caused by *L. major* and *L. tropica* with insights into apoptosis regulation

Taha Masoudsinaki[1,2], Shima Hadifar[1], Hamzeh Sarvnaz[1], Mohammad Farahmand[3], Nasrin Masoudzadeh[1], Vahid Mashayekhi Goyonlo[4], Mohammadali Kerachian[4], Reza Erfanian Salim[5], Mourad Barhoumi[6], Seyed Latif Mousavi Gargari[2*], Hossein Heydari[1*], Sima rafati[1*]

[1] Department of Immunotherapy and Leishmania Vaccine Research, Pasteur Institute of Iran, Tehran, Iran.

[2] Department of Cell Biology, Faculty of Basic Sciences, Shahed University, Tehran, Iran.

[3] Pediatric Infectious Disease Research Center, Tehran University of Medical Sciences, Tehran, Iran.

[4] Cutaneous Leishmaniasis Research Center, Mashhad University of Medical Sciences, Mashhad, Iran.

[5] Noor Eye Hospital, Tehran, Iran.

[6] Molecular Epidemiology and Experimental Pathology (MEEP)/ LR16IPT04, Institut Pasteur de Tunis, Université de Tunis El Manar, Tunisia.

Supplementary File S1. Clinical characteristics of CL lesions due to *L. major* and *L. tropica*.

	Patients number	Sex	Age	Lesion Duration (Month)	Lesion size of punch biopsy (cm^2)	Number of lesions	Location of punch biopsy
L. major	MP1	Male	36	2	17.5	9	shoulder
	MP2	Male	15	3	70	1	Arm
	MP3	Male	19	~1	105	1	Foot
	MP4	Male	41	3-4	1	13	Hand
	MP5	Male	44	1	21	1	Hand
	MP6	Male	73	3-4	6	3	Hand
	MP7	Female	60	5	0.5	1	Face
L. tropica	TP1	Male	30	1	3.5	2	Hand
	TP2	Female	64	5	8	6	Abdomen
	TP3	Female	27	4	4	1	Arm
	TP4	Female	21	7	2	2	Arm
	TP5	Male	24	4	4	3	Hand
	TP6	Male	67	2	2	2	Hand
	TP7	Female	56	4	1	3	Hand
	TP8	Male	29	7	2	1	Foot
	TP9	Male	68	1	2	4	Arm
	TP10	Male	17	1	3	2	Hand

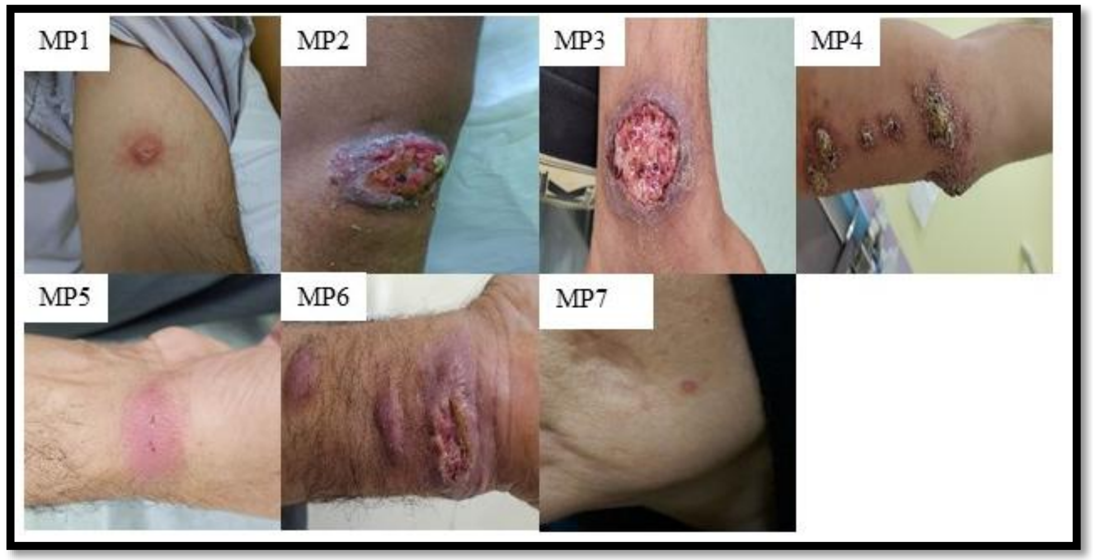

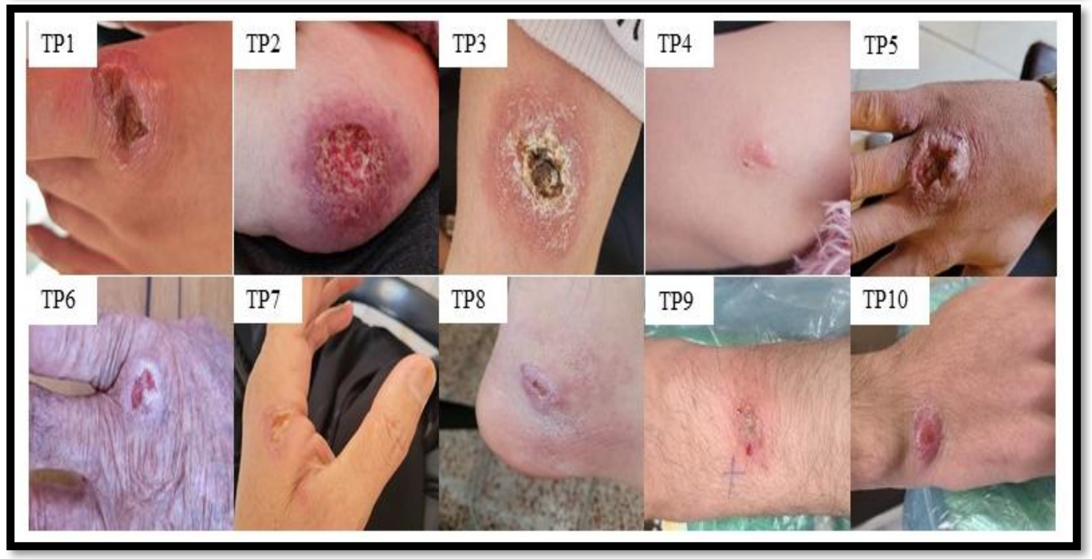

Supplement: Supplementary file 1 — Supplementary Material 1 [file 41598_2025_3802_MOESM1_ESM.pdf]
